# Density functional theory studies on cytosine analogues for inducing double-proton transfer with guanine

**DOI:** 10.1038/s41598-020-66530-8

**Published:** 2020-06-15

**Authors:** Jinjie Xue, Xingping Guo, Xingbao Wang, Yafeng Xiao

**Affiliations:** 1 Molecular genetics laboratory, Children’s Hospital of Shanxi, Women Health Center of Shanxi, Taiyuan, 030013 China; 2 Shanxi Key Laboratory of Birth Defects and Cell Regeneration, Shanxi Population and Family Planning Research Institute, Taiyuan, 030006 China; 3 Training Base of State Key Laboratory of Coal Science and Technology Jointly Constructed by Shanxi Province and Ministry of Science and Technology, Taiyuan University of Technology, Taiyuan, 030024 China; 4 Department of Mathematics, North University of China, Taiyuan, 030051 China

**Keywords:** Density functional theory, DNA, Targeted gene repair

## Abstract

To induce double-proton transfer (DPT) with guanine in a biological environment, 12 cytosine analogues (Ca) were formed by atomic substitution. The DPT reactions in the Watson–Crick cytosine–guanine model complex (Ca_0_G) and 12 modified cytosine–guanine complexes (Ca_1-12_G) were investigated using density functional theory methods at the M06-2X/def2svp level. The intramolecular proton transfers within the analogues are not facile due to high energy barriers. The hydrogen bond lengths of the Ca_1-12_G complexes are shorter than those in the Ca_0_G complex, which are conducive to DPT reactions. The DPT energy barriers of Ca_1-12_G complexes are also lower than that of the Ca_0_G complex, in particular, the barriers in the Ca_7_G and Ca_11_G complexes were reduced to −1.33 and −2.02 kcal/mol, respectively, indicating they are significantly more prone to DPT reactions. The DPT equilibrium constants of Ca_1-12_G complexes range from 1.60 × 10^0^ to 1.28 × 10^7^, among which the equilibrium constants of Ca_7_G and Ca_11_G are over 1.0 × 10^5^, so their DPT reactions may be adequate. The results demonstrate that those cytosine analogues, especially Ca_7_ and Ca_11_, are capable of inducing DPT with guanine, and then the guanine tautomer will form mismatches with thymine during DNA replication, which may provide new strategies for gene therapy.

## Introduction

Genetic mutations, alterations in the DNA sequence, have been identified as the cause of genetic disorders, tumor development and drug resistance in pathogenic microorganisms^1–3^. There are several types of mutations that can occur in DNA, such as copy number variations, duplications, deletions, insertion and single base substitutions, and the latter is the most common type (two-thirds of human genetic diseases are due to single-base alterations)^4^. Therefore, research on the mechanism of point mutations and single-base editing gene therapies has become an attractive topic^4–6^.

**Figure 1:**
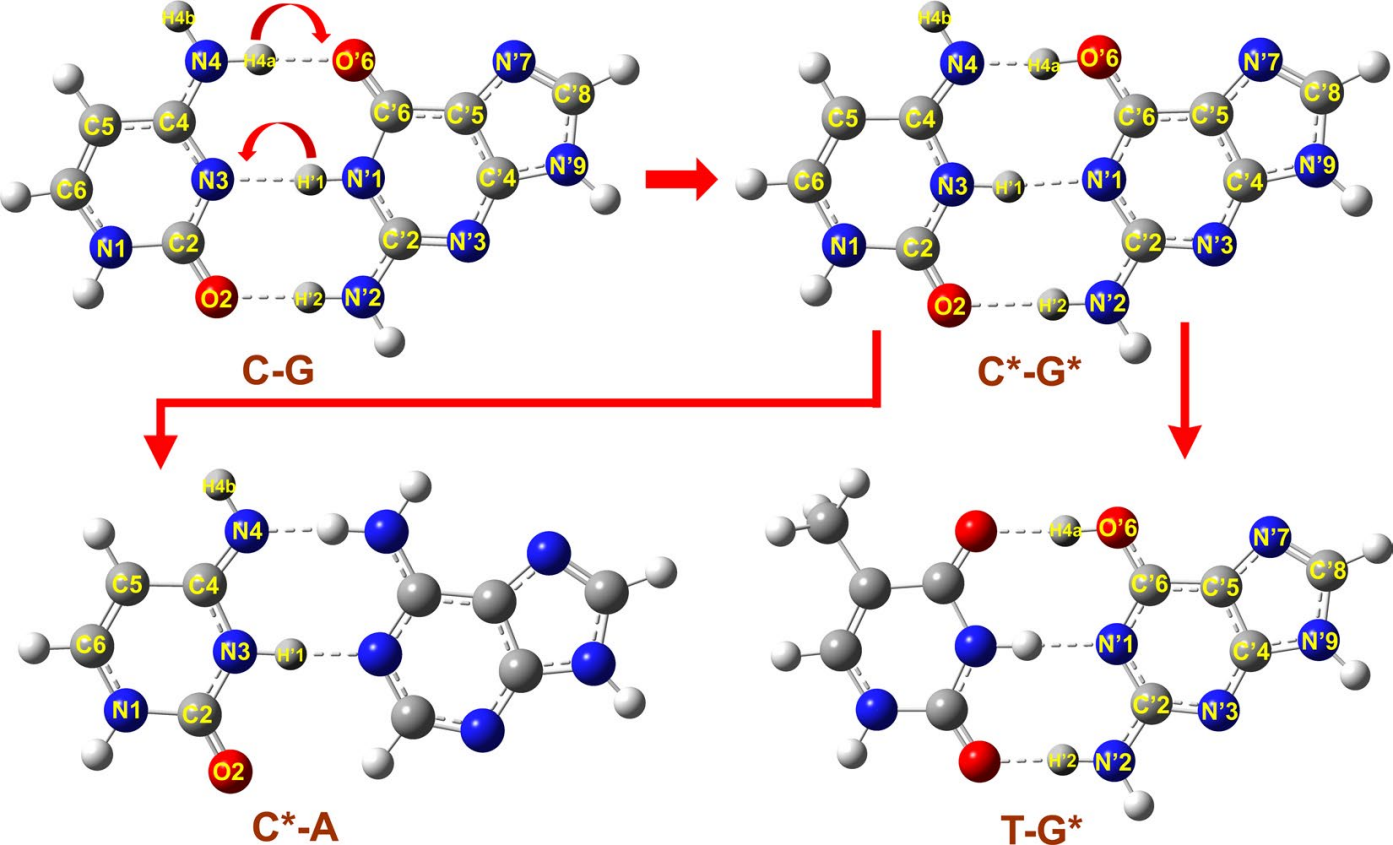
Structures and atomic numberings of the base pairs generated by GaussView 5.0. Top: Canonical C-G base pair and C*-G* base pair resulting from DPT; bottom: tautomers C* and G* mismatched to the bases A and T, respectively.

In double-helical DNA macromolecules, the complementarity between pyrimidines (cytosine and thymine) and purines (guanine and adenine) are due to hydrogen bonds. Each hydrogen bonding is essentially a proton trapped in an asymmetric double well potential between two electronegative atoms^7^. It has been hypothesized that proton transfer within the double well potential is an important mechanism of base substitution^7,8^. Tautomerism in guanine–cytosine base pairs resulting from intermolecular proton transfer has been suggested to be responsible for the universal guanine–cytosine to adenine–thymine mutation frequently observed in bacteria, fungi, plants, and animals^9^. The tautomers, indicated by an asterisks (C* and G*), resulting from the transfer of cytosine H_4a_ to guanine and the back transfer from guanine H’_1_ to cytosine cannot form hydrogen-bonded complexes with their natural counterparts but can form mismatches with the bases A and T, respectively, during DNA replication (Fig. 1)^7,10^. Under normal circumstances, the relatively high energy barrier within the double well potential prevents the forward double-proton transfer (DPT) reaction, which accounts for the stability of DNA and the fidelity with which the genetic code is preserved and transmitted to daughter cells^11,12^. However, when the quantum nature of the proton is taken into account, the proton has a small yet finite probability of tunneling through the barrier^12^. When such double proton tunneling occurs during DNA synthesis, single base substitutions will occur. Fu *et al*. showed that among several possible DNA mutation processes, only the DPT mechanism could fully explain the universal mutation bias^9^.

Previous studies have focused on various environmental factors that might facilitate intermolecular proton transfer within base pairs. Zhang, J. D. *et al*. showed that when an additional hydride is placed on the C_6_ and C_4_ positions of cytosine, the anionic complex formed, which facilitates guanine H_1_ transfer to the N_3_ site of cytosine^13^. Noguera, M. *et al*. found that protonation at the N_7_ and O_6_ sites of guanine, affording H^+^ G_N7_C and H^+^G_O6_C, strengthens the binding of the base pair and facilitates the N_1_–N_3_ single-proton-transfer reaction^14^. The influences of metal cation (Cu^+^, Ca^2+^ and Cu^2+^) coordination to the CG and AT base pairs on intermolecular proton transfer were studied using density functional theory (DFT) methods^15,16^. Alya, A. A. *et al*. investigated the DPT reaction of a GC base pair under the effect of uniform electric fields on the order of 10^8^ to 10^9^ Vm^−1^. They considered that fields applied along the axis of the double proton transfer in the -x (defined in the C to G direction) direction favor the canonical over the rare tautomers^12^. Chen, H. Y. *et al*.^17^ showed that the substantial effect of GC stacking originates from the electrostatic interactions between the dipoles of the outer GC base pairs and the middle GC^•−^ base-pair radical anion, the extent of the charge delocalization is very small and has little effect on proton transfer in GC^•−^. The effect of the surrounding water molecules on the DPT in GC was investigated using DFT methods, and the results demonstrate that water is crucial to the proton reactions. It does not act as a passive element but actually catalyzes the DPT^18^.

Thus far, few studies have examined the effects of neutral cytosine analogues on the DPT reaction in CG base pare. The purpose of this study was to use existing physic-chemical understanding and predictive capability to identify neutral cytosine analogues that can induce DPT reactions with guanine under physiological conditions and provide new strategies for gene therapy.

## Results and Discussions

### Molecular structures of the modeled cytosine analogues

**Figure 2:**
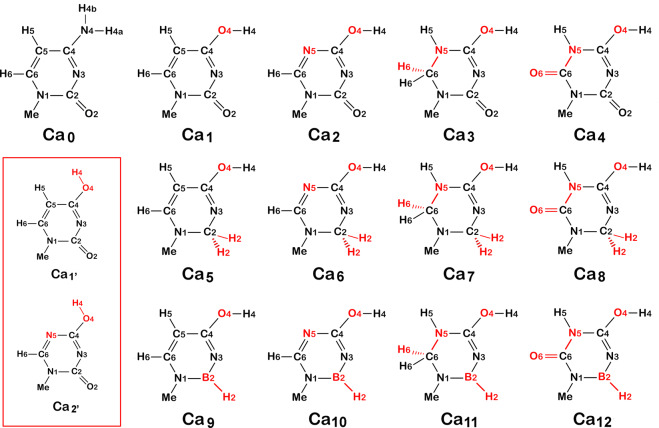
Molecular structures of the modeled cytosine analogues (Ca). The atoms that had being replaced were shown in red. The box showing the conformers of Ca_1_ and Ca_2_.

To provide minimized structures to mimic the Watson–Crick base pairing in duplex DNA or RNA, the sugar-attachment sites of cytosine and guanine were methylated^19^. To build a library of cytosine analogues, multiple atoms on the cytosine were replaced. To improve its ability to donate protons, the amino group at C_4_ was replaced by a hydroxy moiety. To improve the ability of N_3_ to accept protons, the carbonyl oxygen at C_2_ was replaced by a hydrogen, and separately, the C_2_ carbon atom was replaced by a boron atom. In addition, C_5_ was replaced by a nitrogen atom. The carbon atom at C_6_ was replaced with methine, methylene, and carbonyl moieties. A total of 12 cytosine analogues were modeled (Fig. 2).

### Energy barriers and rate constants of the intramolecular proton transfer

First, the structures of all the monomers, Ca_0-12_, were optimized by DFT M06-2X/def2svp method. The vibration analysis showed no imaginary frequencies in those monomers, indicating that the structures are stable at the current calculation level. Because both the O_4_ atom’s ability to provide protons and the N_3_ atom’s ability to acquire them are enhanced, the modeled cytosine analogues may undergo intramolecular proton transfer (H_4_ transfer from O_4_ to N_3_). Therefore, the intramolecular single proton transfer (SPT) reactions of all monomers were investigated. The calculated SPT energy barriers (Δ*G*^≠^) within the cytosine analogues ranged from 24.51 to 32.23 kcal/mol (Table 1). The forward reaction rate constants (calculated from Δ*G*^≠^) of the intramolecular proton transfer process range from 3.40 × 10^−5^ to 1.23 × 10^−10^ s^−1^, which indicate that the intramolecular SPT process are not facile. Therefore, the barriers of all cytosine analogues are relatively high for the proton to hop, indicating that these molecules can be used as candidate molecules to induce DPT reactions with guanine.

**Table 1:** The values of the barrier of the intramolecular SPT reactions (Δ*G*^≠^), the energy difference between analogues and their conformers (ΔE), the forward and reverse barriers of the intermolecular DPT reactions (Δ*G*^≠^_*f*_ and Δ*G*^≠^_*r*_), the forward and reverse reaction rate constants of intermolecular DPT reactions (k_f_ and k_r_), and the equilibrium constants (K_eq_). All energy barrier values are given in kcal/mol.

Ca_*n*_	Δ*G*^≠^	ΔE	Δ*G*^≠^_*f*_	Δ*G*^≠^_*r*_	k_f_ (s^−1^)	k_r_ (s^−1^)	K_eq_
Ca_0_	43.13	—	8.31/8.80/7.60	0.22/0.71/−0.49	4.05 × 10^6^	2.04 × 10^12^	1.99 × 10^−6^
Ca_1_	30.71	−2.99	1.07	1.60	1.14 × 10^12^	4.81 × 10^11^	2.37 × 10^0^
Ca_2_	32.23	−0.49	0.87	1.16	1.57 × 10^12^	9.83 × 10^11^	1.60 × 10^0^
Ca_3_	28.19	−4.45	−1.20	3.79	4.53 × 10^13^	1.38 × 10^10^	3.28 × 10^3^
Ca_4_	31.50	−4.59	−1.54/−0.49/−1.15	−0.08/0.97/0.31	1.43 × 10^13^	1.34 × 10^12^	1.07 × 10^1^
Ca_5_	29.86	−2.97	−0.62	4.24	1.77 × 10^13^	6.63 × 10^9^	2.67 × 10^3^
Ca_6_	31.10	−0.47	−0.35	3.67	1.14 × 10^13^	1.67 × 10^10^	6.83 × 10^2^
Ca_7_	28.61	−4.27	−1.33	6.01	5.60 × 10^13^	3.75 × 10^8^	1.49 × 10^5^
Ca_8_	31.68	−4.55	−0.48	4.95	1.41 × 10^13^	2.09 × 10^9^	6.75 × 10^3^
Ca_9_	27.13	−3.23	0.00	5.07	6.46 × 10^12^	1.72 × 10^9^	5.09 × 10^3^
Ca_10_	28.19	−0.52	−0.20	5.22	8.94 × 10^12^	1.35 × 10^9^	6.62 × 10^3^
Ca_11_	24.51	−4.86	−2.02	8.06	1.72 × 10^14^	1.34 × 10^7^	1.28 × 10^7^
Ca_12_	28.36	−4.89	−0.74	5.62	2.15 × 10^13^	7.06 × 10^8^	3.05 × 10^4^

Due to the rotational orientation of the hydroxyl group, cytosine analogues have possible conformers in which the H_4_ atom is oriented toward the C_5_ or N_5_ atom, as shown in Fig. 2. The conformers were optimized by DFT at the current level and no imaginary frequencies were found. The present calculations show all the analogues have lower energy compared with their conformers (Table 1), indicating that these analogues are more stable than theirs conformers.

### The vdW surfaces and ESP extrema of the cytosine analogues

**Figure 3:**
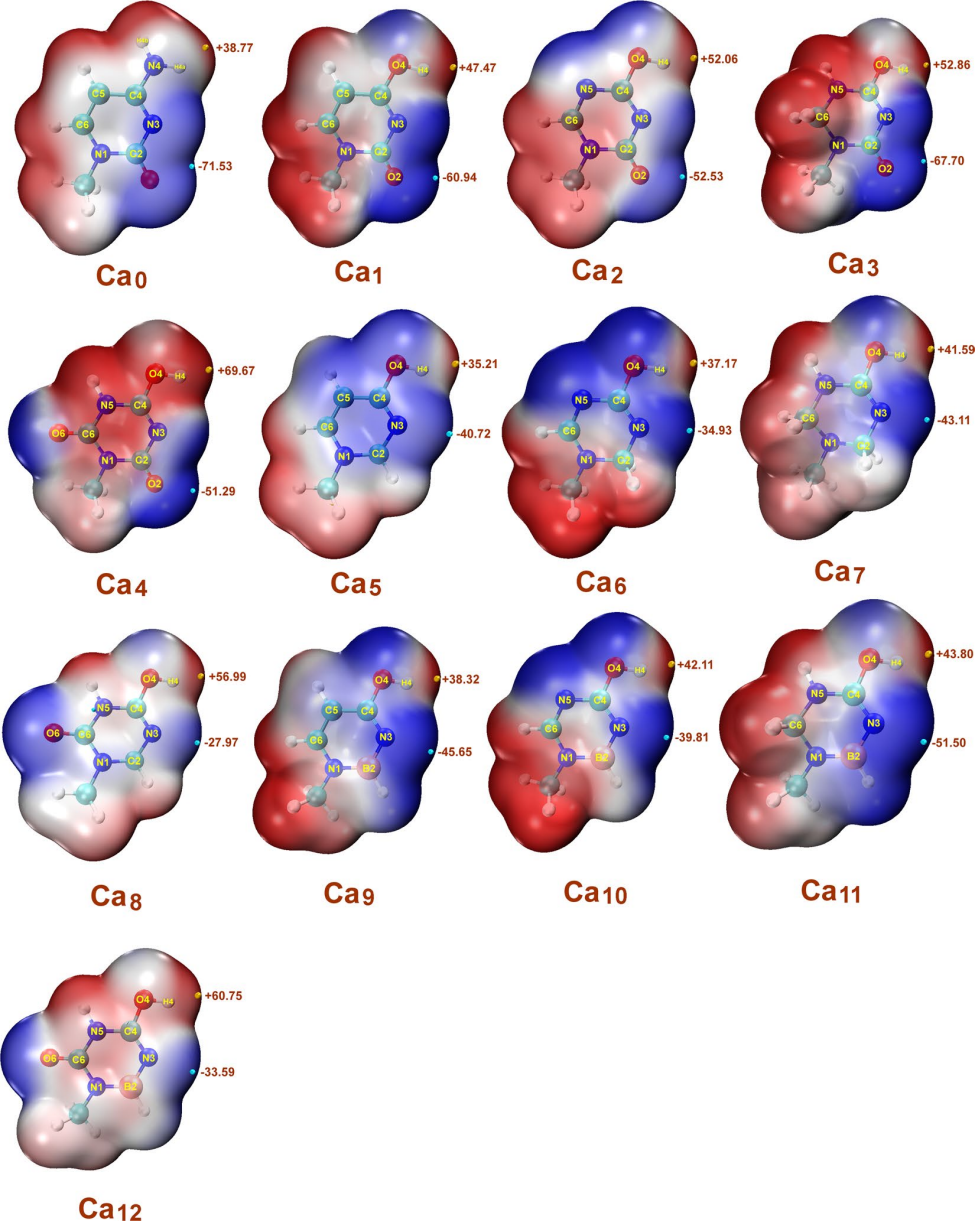
ESP-mapped vdW surfaces and ESP extrema of cytosine analogues generated by Multiwfn 3.6 and VMD 1.9.1 (http://www.ks.uiuc.edu/Research/vmd/). The red color indicates positive ESP regions on the vdW surface; the blue regions correspond to negative ESP regions of the vdW surface. The significant local maxima and minima of ESP on the vdW surfaces are represented as orange and cyan spheres, and labelled by values given in kcal/mol.

The electrostatic potential (ESP)-mapped van der Waals (vdW) surface has been used extensively for interpreting and predicting reactivity and intermolecular interactions of a wide variety of chemical systems^20,21^. The negative charge of the Ca_0_ molecule is concentrated on the O_2_ atom with an ESP extrema value of −71.53 kcal/mol. The positive electrostatic potential is centered around H_4a_ of the amino group with an ESP extrema value of +38.77 kcal/mol (Fig. 3. Ca_0_). When the amino group at the C_4_ position of the cytosine was replaced by a hydroxy moiety, the ESP extrema value was increased to +47.47 kcal/mol, which is favorable for nucleophilic attack and release of the proton (Fig. 3. Ca_1_). Furthermore, when the C_5_ carbon atom was replaced by a nitrogen atom and the carbon atom at C_6_ was a methine, methylene, or carbonyl, the ESP extrema value of H_4_ was increased to +52.06 kcal/mol, +52.86 kcal/mol and +69.67 kcal/mol, respectively, which are all favorable for nucleophilic attack and release of the proton (Fig. 3. Ca_2_-Ca_4_). When the carbonyl oxygen at C_2_ was replaced by a hydrogen, the negative charges and the ESP extrema shift to the N_3_ atom, which is favorable for electrophilic attack and accepting a proton at the N_3_ site (Fig. 3. Ca_5_-Ca_8_). Furthermore, when the C_2_ carbon atom was replaced by a boron atom, the ESP extrema values of N_3_ are strengthen to −45.65 kcal/mol, −39.81 kcal/mol, −51.50 kcal/mol and −33.59 kcal/mol, which are all more favorable for electrophilic attack and accepting a proton at the N_3_ site (Fig. 3. Ca_9_-Ca_12_). So, these atomic substitutions, especially the substitutions of a hydroxy moiety for the amino group, a hydrogen for the carbonyl oxygen at C_2_ and a boron atom for the C_2_ carbon atom, are all favorable to induce DPT reactions with guanine.

### Energy barriers and equilibrium constants of the DPT reaction

The structures of all the Ca_n_G complexes were optimized by DFT at the current level. The vibration analysis showed no imaginary frequencies in these complexes, indicating that the structures are stable. Intermolecular DPT, which generates hydrogen-bonded pairs of tautomers, was computationally predicted for all the complexes. DPT can take place through a concerted DPT (Ca_1-3_G and Ca_5-12_G) or via a stepwise mechanism involving two distinct SPT steps (Ca_0_G and Ca_4_G). The vibration analysis of each proton transfer reaction showed one imaginary frequency, and the vibration mode of the imaginary frequency corresponds to the reactants and products assigned to the transition state. An intrinsic reaction coordinate was prepared to confirm the existence of the transition states.

The Gibbs free energies of the fully optimized Ca_0_G complexes were defined as having zero energy. The relative Gibbs free energies of the optimized reactants, the first and second single-proton transition states, the double-proton transferred states, the intermediate product of single proton transfer, and the product of double-proton transfer were calculated.

The Ca_0_G complex, the model of the Watson–Crick guanine–cytosine (GC) base pair, undergoes a stepwise DPT process. The dissociation energy (DE) of the Ca_0_G base pair at the current level of theory is 21.74 kcal/mol, which is in good agreement with the reported experimental value of 21.0 kcal/mol^22^. However, the previously calculated DE values were 25.4 kcal/mol^23^, 23.8 kcal/mol^24^, and 24.4 kcal/mol^25^. This result indicates that the present calculations are a better mimic of physiological conditions. The energy of the DPT product of Ca_0_G lies 9.15 kcal/mol above the reactant species, which is similar to previously calculated values of 9.8 kcal/mol^14^ and disfavors the DPT reaction. The Ca_0_G–Ca_0_*G* equilibrium (with a calculated equilibrium constant of 1.99 × 10^−6^, which is in good agreement with previously estimated values of 2.0 × 10^−6^)^12^ largely favors the reactants, so this double-proton-transfer reaction will rarely occur. In fact, we also calculated the dissociation energy of Ca_0_G and the equilibrium constant of the DPT reaction using the DFT B3LYP/6-311 + +G(d,p) method. And the calculated values were 13.25 kcal/mol and 4.53 × 10^−8^ respectively, which were quite different from the experimental values (21.0 kcal/mol) of the dissociation energy and the previously estimated values (2.0 × 10^−6^) of the equilibrium constant. So, these results calculated by the DFT M06-2X/def2svp method are in good agreement with previous findings related to the DPT reaction of GC base pairs, indicated the calculations are more reasonable.

The forward DPT free energy barriers of the 12 analogue complexes (Δ*G*^≠^_*f*_) range from −2.02 to 1.07 kcal/mol, which are significantly lower than the value of Ca_0_G (8.80 kcal/mol). The barriers increased in the order Ca_11_G < Ca_7_G < Ca_3_G < Ca_12_G < Ca_5_G < Ca_4_G < Ca_8_G < Ca_6_G < Ca_10_G < 0 < Ca_9_G < Ca_2_G < Ca_1_G<<Ca_0_G. Lower energy barriers favor the DPT reaction, especially for complexes with negative barriers. The forward rate constants (calculated from Δ*G*^≠^_*f*_) of the DPT process range from 1.14 × 10^12^ to 1.72 × 10^14^ s^−1^, which are significantly higher than the value of Ca_0_G (4.05 × 10^6^ s^−1^). The reverse DPT free energy barriers of the 12 analogue complexes range from 0.97 to 8.06 kcal/mol, which are higher than the value of Ca_0_G (0.71 kcal/mol). In addition, the reverse rate constants (calculated from Δ*G*^≠^_*r*_) of the DPT process range from 1.34 × 10^7^ to 1.34 × 10^12^ s^−1^, which are lower than the value of Ca_0_G (2.04 × 10^12^). The DPT equilibrium (k_eq_ = k_f_/k_r_) ranges from 1.60 × 10^0^ to 1.28 × 10^7^, which is significantly higher than the value of Ca_0_G (1.99 × 10^−6^). The DPT equilibrium constants increased in the order Ca_0_G<<0 < Ca_2_G < Ca_1_G < Ca_4_G < Ca_6_G < Ca_5_G < Ca_3_G < Ca_9_G < Ca_8_G < Ca_10_G < Ca_12_G < Ca_7_G < Ca_11_G. For all 12 analogue complexes with positive equilibrium constants, the reaction may occur in the DPT direction. In particular, the DPT equilibrium constants of Ca_7_G and Ca_11_G (1.49 × 10^5^ and 1.28 × 10^7^) are over 1.0 × 10^5^, which indicates that these two proton transfer reactions will occur adequately. All the values are shown in Table 1.

### Geometries of the DPT reactions of the analogue complexes

In the Ca_0_G complex, the intermonomer N_3_ − N′_1_ distance (as labeled in Fig. 1) is 2.92 Å. This distance is consistent with the calculated distance (2.95 Å) and the experimental distance (3.09 Å)^12^, as is the distances of N_4_ − O′_6_. As the reaction progresses from the reactant to transition state 1 (TS_1_), the intermediate product of the single proton transfer (SPT) and TS_2_, the guanine and cytosine monomers approach each other. The N_3_ − N′_1_ distances in TS_1_, SPT and TS_2_ are 2.65 Å, 2.72 Å and 2.78 Å, respectively. As the reaction proceeds, the N′_1_ − H′_1_ bond stretches faster than the N_4_ − H_4a_ bond and completes the proton transfer first. In the products, the N_3_ − N′_1_ bridge elongates again to 2.86 Å, which is close to their original separation in the reactants.

In the Ca_1-12_G analogue complexes, the intermonomer N_3_ − N′_1_ distance and O_4_-O′_6_ distance range from 2.70 to 2.81 Å and 2.46 to 2.61 Å, respectively, which are all shorter than those in the Ca_0_G complex (2.92 Å and 2.85 Å), indicating that the forward DPT reaction of those complexes is more favorable than that of the Ca_0_G complex. As the reaction progresses from the reactant to TS, most of the N_3_ − N′_1_ distances and O_4_-O′_6_ distances are shorter than the distance in the Ca_0_G complex and favor the DPT reaction. In the products, the N_3_ − N′_1_ and O_4_-O′_6_ bridges elongate again, and more than half of these bridges are longer than the distance in the Ca_0_G DPT product, which indicates that the DPT reverse reaction in those complexes is more difficult than it is in the Ca_0_G DPT product (Table 2).

**Table 2:** Distances (in Å) between the electronegative atoms involved in the two H-bonds in Ca_n_G during the DPT process.

Ca_n_G	Electronegative Atoms	Reactant	TS (TS_1_/SPT/TS_2_)	Product
Ca_0_G	N_3_-N′_1_	2.92	2.65/2.72/2.78	2.86
	N_4_-O′_6_	2.85	2.62/2.59/2.48	2.58
Ca_1_G	N_3_-N′_1_	2.81	2.61	2.84
	O_4_-O′_6_	2.54	2.43	2.59
Ca_2_G	N_3_-N′_1_	2.79	2.60	2.82
	O_4_-O′_6_	2.52	2.46	2.61
Ca_3_G	N_3_-N′_1_	2.77	2.61	2.87
	O_4_-O′_6_	2.50	2.43	2.60
Ca_4_G	N_3_-N′_1_	2.76	2.71/2.69/2.60	2.81
	O_4_-O′_6_	2.46	2.40/2.45/2.50	2.65
Ca_5_G	N_3_-N′_1_	2.74	2.57	2.92
	O_4_-O′_6_	2.61	2.52	2.52
Ca_6_G	N_3_-N′_1_	2.80	2.56	2.94
	O_4_-O′_6_	2.60	2.49	2.53
Ca_7_G	N_3_-N′_1_	2.72	2.57	2.93
	O_4_-O′_6_	2.58	2.49	2.51
Ca_8_G	N_3_-N′_1_	2.78	2.56	2.89
	O_4_-O′_6_	2.52	2.41	2.59
Ca_9_G	N_3_-N′_1_	2.78	2.59	2.94
	O_4_-O′_6_	2.58	2.45	2.54
Ca_10_G	N_3_-N′_1_	2.78	2.58	2.91
	O_4_-O′_6_	2.56	2.44	2.57
Ca_11_G	N_3_-N′_1_	2.70	2.59	2.95
	O_4_-O′_6_	2.53	2.47	2.55
Ca_12_G	N_3_-N′_1_	2.77	2.65	2.91
	O_4_-O′_6_	2.49	2.40	2.62

### Effects of cytosine atom substitution on the DPT reaction

When the amino group at the C_4_ position of the cytosine was replaced by a hydroxy moiety and the other conditions were held constant, the energy barrier of the DPT reaction decreased by 7.73 kcal/mol. This result indicates that the hydroxy group significantly favored the DPT reaction. Since the electronegativity of oxygen atom is lower than that of nitrogen atom, the attraction of oxygen to proton is less than that of nitrogen, which is the possible reason that favors the transfer of H_4_ from O_4_ to N′_6_.

When the C_2_ carbonyl oxygen atoms of the cytosine analogues were replaced by hydrogen, the energy barriers of the DPT decreased by an average of 1.01 kcal/mol. Replacing C_2_ with a boron atom further decreased the energy barriers of the DPT by an average of 0.06 kcal/mol, further favoring the DPT reaction. Removing the carbonyl group from C_2_ and replacing C_2_ with a less electronegative boron atom can transfer the negative charge to N_3_, which is conducive to the ability of N_3_ to acquire proton and favors the transfer of H’_1_ from N′_1_ to N_3_. A large number of BN-containing heterocycles have been developed by means of the substitution of a carbon-carbon double bond with an isoelectronic boron-nitrogen bond (BN-substitution)^26,27^. BN-substituted nucleobases are very attractive BN-substituted heterocyclic compounds. Bielawski *et al*.^28^ reported the synthesis of B(6)-phenyl-BN-uracil, which was characterized by mass spectrometry, IR spectroscopy and elemental analysis. It is interesting to note that Hiroshi *et al*.^26^ also reported the synthesis of B(6)-substituted 5-aza-6-borauracils (BN-substituted uracils) and -thymines (BN-substituted thymines). The structures of these BN-substituted nucleobases are similar to the cytosine analogues in present study. Therefore, these boron-containing cytosine analogues can be synthesized as realistic candidates.

When the C_5_ carbon atom of the cytosine analogues was replaced by a nitrogen atom, the energy barriers of the DPT decreased by an average of 0.03 kcal/mol. When the double bonds between N_5_ and C_6_ of these analogues were hydrogenated to single bonds, the energy barriers of the DPT further decreased by an average of 1.62 kcal/mol. However, when the hydrogen on C_6_ was exchanged for a carbonyl oxygen, the energy barriers of the DPT increased again. Therefore, the second result seemed to indicate that the DPT reaction was favored. Replacing C_5_ with highly electronegative nitrogen atom may transfer the negative charge from O_4_ to N_5_, which will reduce the attraction of O_4_ to proton and favors the transfer of H_4_ from O_4_ to N′_6_.

## Conclusions

The abilities of 12 modified cytosine–guanine complexes to undergo DPT were predicted and compared using theoretical calculations. The DE value, DPT barriers, hydrogen bond lengths and equilibrium constants of the Ca_0_G complex are similar to previously calculated values and experimental values, indicating that the present calculation method is reasonable. Because of the intramolecular SPT process are not facile, the 12 modeled cytosine analogues can be used as candidate molecules to induce DPT reactions with guanine. Eight modified complexes (Ca_3_G, Ca_5_G and Ca_7-12_G) were significantly more prone to undergo DPT reactions than Ca_0_G. In particular, Ca_7_G and Ca_11_G may undergo sufficient DPT reactions under physiological conditions according to the present calculations. Here, we present the part of the study on theoretical calculations and predictions of candidate molecules. Next these analogues are expected to be synthesized for incorporation into single-stranded RNAs and to be validated in *in-vivo* model. By binding such modified RNA to DNA or RNA, DPT reactions of guanine may be induced at a fixed point. If cytosine analogues that can spontaneously induce DPT reactions of guanine under physiological conditions are identified experimentally, targeted pathogenic mutations can be used to restore original functions at the level of DNA or RNA.

## Theoretical Methods

DFT with the M06-2X functional and the def2svp basis set has been the primary research method used to investigate proton transfer reactions between cytosine analogues and guanine. The M06-2X functional is a high nonlocality functional with twice the nonlocal exchange (2X), and it is considered suitable for the study of main-group thermochemistry, thermochemical kinetics, noncovalent interactions, and electronic excitation energies to the valence and Rydberg states. The M06-2X functional also gives the best performance for hydrogen-transfer barrier calculations and the lowest values of balanced mean unsigned error (BMUE), which means it gives the best overall performance for barrier calculations^29–31^. The D3 dispersion correction was used in this study to improve the accuracy of the energy and structure calculations^32–35^. To simulate the DNA surroundings in a biological environment, all the calculations were carried out with water solvation at *T* = 310.15 K (37 °C) and *p* = 1 atm. The sophisticated polarizable continuum model^36^ has been used to investigate solute–solvent interactions in water using the scrf = (solvent = water, pcm) keyword. The Gaussian 09 program package was used throughout this study^37^. The structures of all the monomers and complexes were optimized by DFT at the current level. The vibration analyses were performed to determine whether the molecular structures were stable. The H_4_ and H’_1_ hydrogen atoms of the optimized complexes were placed in the middle points between two electronegative atoms (N_3_-N′_1_, N_4_-O′_6_ or O_4_-O′_6_) and then the structures of the transition states were optimized using the opt = (calcall, ts, noeigen) keyword. The vibration analyses were performed to determine the existence of the transition states. The forward energy barrier, Δ*G*^*≠*^_*f*_, is the difference between the Gibbs free energy of the transferred states and the reactants. The reverse energy barrier, Δ*G*^≠^_*r*_, is the difference between the Gibbs free energy of the transferred states and the products.

The GaussView 5.0^38^ was used to create molecular structures of the modeled cytosine analogues. The molecular structure of classic cytosine, at first, was created and optimized by the program package. And then the 12 modeled cytosine analogues were created by replacing the atoms in the classic cytosine and optimized by the program package.

To investigate the ability of N_3_ to accept protons and O_4_ to donate protons, the ESP-mapped vdW surfaces and ESP extrema were rendered using the VMD 1.9.1 program^39^ based on the outputs from the Multiwfn 3.6 program^40,41^.

To investigate the equilibrium of the DPT reactions and chemical reactions of the analogue complexes, the forward and reverse rate constants (k_f_ and k_r_) were given by the following equation^42^

1 $$k=2.1\times {10}^{10}T{{\rm{e}}}^{-1000\varDelta {G}^{\ne }/(1.9859\times T)}$$

Where k is the forward or reverse rate constants, *T*=310.15 K (37 °C), Δ*G*^≠^ is the forward or reverse energy barriers. The equilibrium constants (K_eq_ values) of the DPT reactions are the quotients of k_f_ and k_r_ (K_eq_= k_f_/k_r_).
